# Short-read genome sequencing allows ‘en route’ diagnosis of patients with atypical Friedreich ataxia

**DOI:** 10.1007/s00415-023-11745-8

**Published:** 2023-04-29

**Authors:** Zofia Fleszar, Claudia Dufke, Marc Sturm, Rebecca Schüle, Ludger Schöls, Tobias B. Haack, Matthis Synofzik

**Affiliations:** 1 Department of Neurodegenerative Diseases and Hertie Institute for Clinical Brain Research, University of Tübingen, Tübingen, Germany; 2 German Centre for Neurodegenerative Diseases (DZNE), Tübingen, Germany; 3 Institute of Medical Genetics and Applied Genomics, University of Tübingen, Tübingen, Germany; 4 Division of Neurodegenerative Diseases, Department of Neurology, Heidelberg University Hospital, Heidelberg, Germany; 5 Centre for Rare Diseases, University of Tübingen, Tübingen, Germany

## Introduction

Identification of the molecular cause is complicated in hereditary ataxias not only by the pleiotropy of underlying > 100 ataxia genes [9, 15, 16], but also by the broad phenotypic variability—even for the same gene [11]—and the various underlying mutational mechanisms. Compared to other genetic neurological diseases, this variety of mutation mechanisms includes a particularly high number of different short-tandem repeat-expansions (STRs) in ataxias, in both coding (e.g. PolyQ SCAs [5]) and non-coding regions (e.g. Friedreich Ataxia [FA] [3], *RFC1* [4], GAA-*FGF14* [12], *GLS *[6]).

So far, pre-selection of the correct genetic diagnostic method according to the presumed underlying mutational mechanism has been key in current ataxia routine diagnostics: STRs are missed by standard next-generation sequencing (NGS) panel-based approaches; and while exome sequencing (ES)-based STR analyses [18] are now being considered standard for clinical routine, they still show only moderate specificity for exonic STRs [18] and—by design—fail almost completely for most of the intronic STRs in ataxias. This pre-selection of genetic diagnostics, e.g. direct fragment length analysis for FA, however, is led by clinical suspicion based on the main clinical phenotype and additional relevant information (such as e.g. the supposed inheritance mode). However, this pre-selection is vulnerable to bias caused by clinical reasoning based on typical phenotypic presentations or false interpretation of family history, which may result in missing out on the correct diagnosis by excluding the pertinent genetic test in case of atypical phenotypic presentations or misleading prior interpretations. By a series of three distinct cases of atypical FA, we here showcase how the introduction of short-read genome sequencing (SR-GS) allows to overcome these biases in the work-up of complex ataxias, as it allows to detect even intronic STRs (i) “en route”, i.e. with detection not requiring any primary direct gene analysis; and (ii) in a phenotype-independent fashion”, i.e. also for those atypical phenotypic presentations where the corresponding gene and mutational mechanism had not been part of the prior differential clinical diagnosis.

## Methods

A consecutive series of 127 ataxia subjects was recruited by the ataxia outpatient clinics of the Center of Neurology, Tübingen between 2021 and 2022 and investigated by SR-GS. Sequencing libraries were generated using the Illumina DNA PCR-Free protocol and sequenced on a Illumina NovaSeq 6000 sequencer with a target depth of 38x. Sequencing reads where mapped to the GRCh38 reference genome using BWA-mem2 v.2.2.1 (https://github.com/bwa-mem2/bwa-mem2) and repeat expansions where detected with ExpansionHunter v5.0.0 (https://github.com/Illumina/ExpansionHunter). Patients with unexpected biallelic pathological GAA expansions in *FXN* received in-depth phenotyping by imaging and electrophysiological studies in addition to physical examination. The Institutional Review Board of the University of Tübingen approved the study (AZ 598/2011BO1). All subjects provided written informed consent before participation and publication according to the Declaration of Helsinki.

## Results

SR-GS allowed to identify three ataxia subjects with biallelic GAA expansions in *FXN* where FA had not been part of the initial differential diagnosis.

**Fig. 1:**
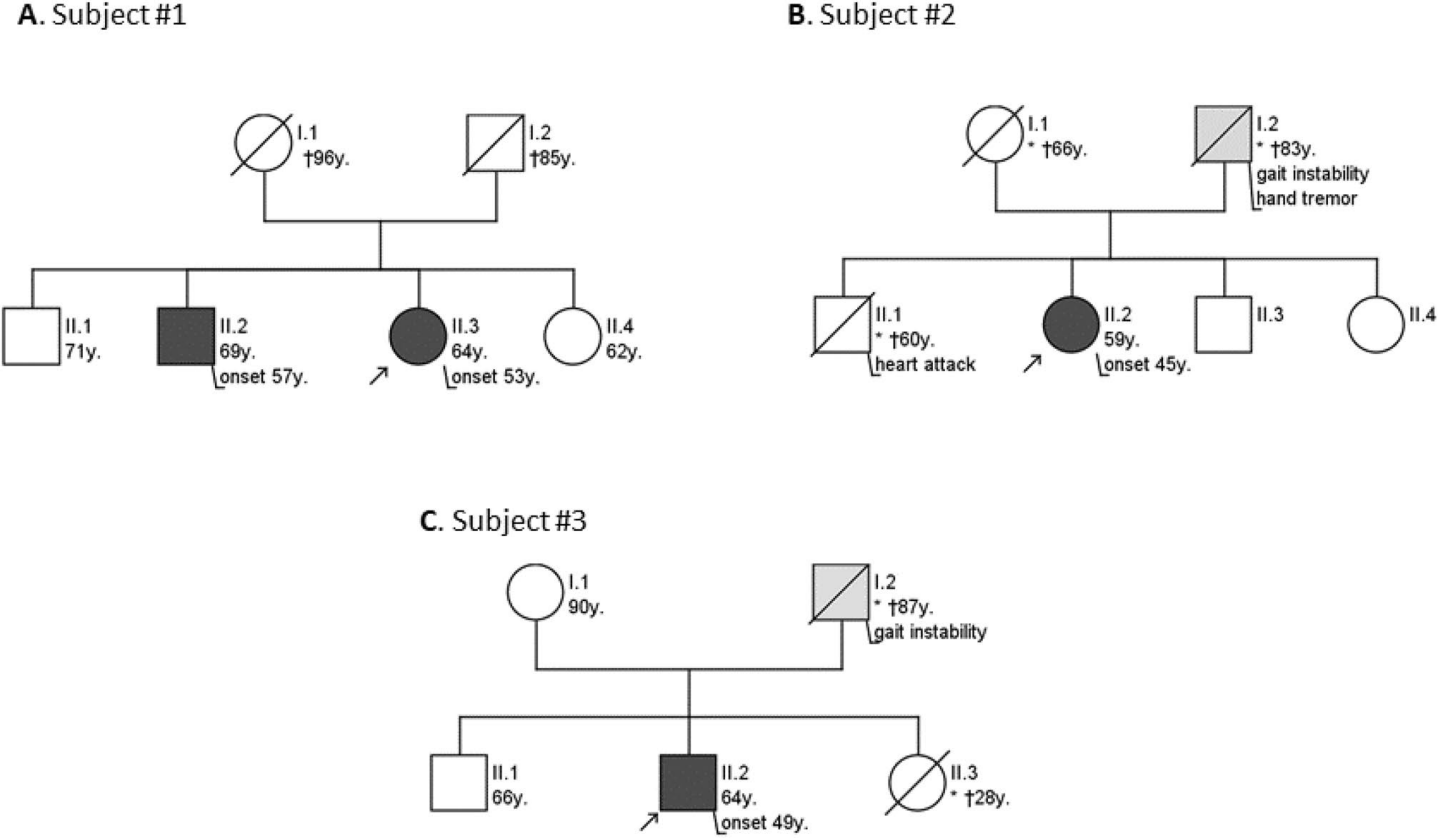
Pedigrees of the 3 subjects with atypical FA presentations. Given the progressive gait disturbances in the parental generation of subject #2 and subject #3, an autosomal dominant mode of inheritance of the ataxia was initially assumed for both subjects

**Fig. 2:**
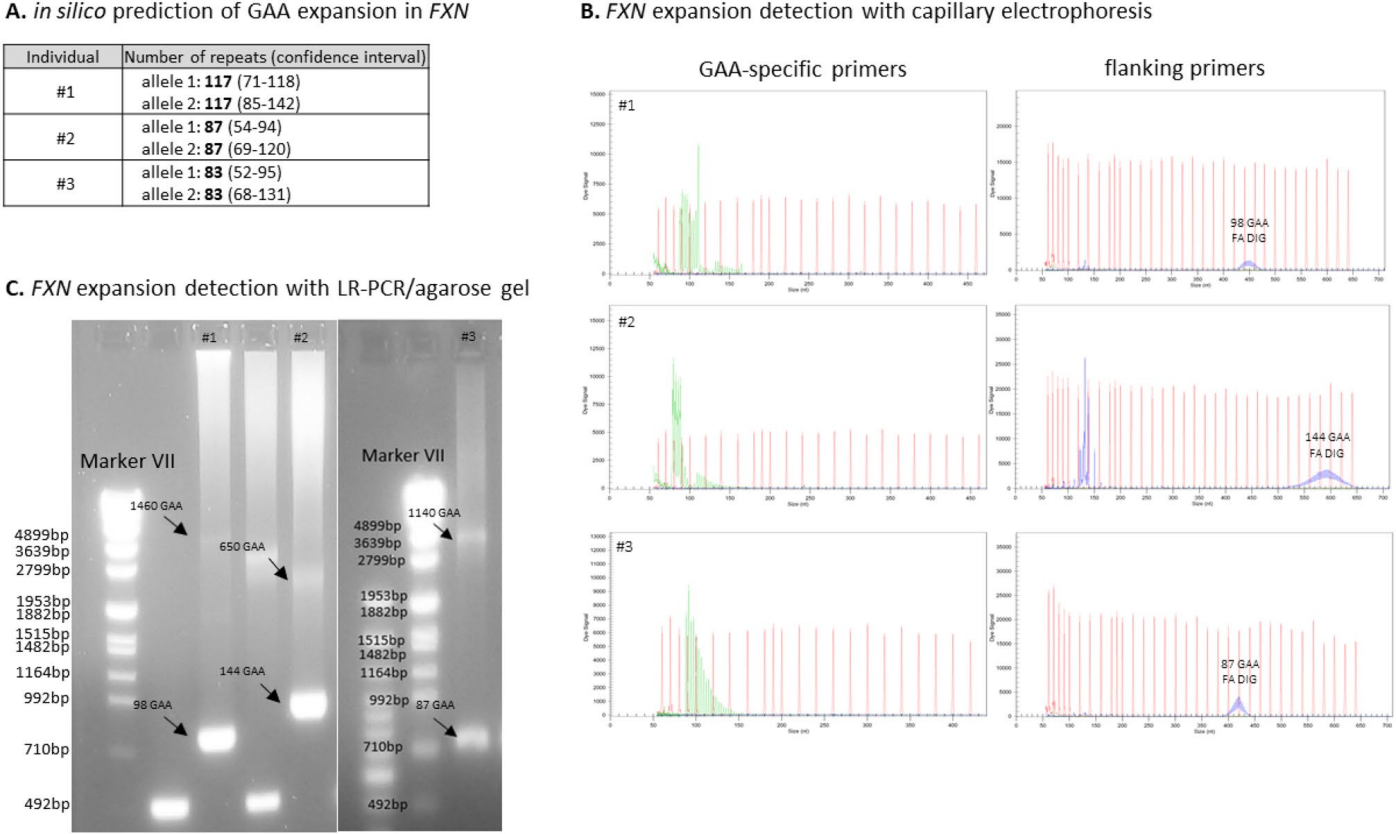
**A** in silico prediction of an expanded size of the GAA repeat motive in *FXN* (hg38, chr9:69037286-69037304) using ExpansionHunter v5.0.0. While — given that the size of the expanded repeat exceeds the SR-GS read length - exact quantification of the full repeat size is not possible, it allows to predict an at least minimal repeat length, with these results suggesting the presence of a biallelic repeat expansion (number of repeats for both alleles) in all 3 patients. **B** Fragment length analysis (FLA) showing the decremental sawtooth pattern with the GAA-specific primers (left). The respective shorter allele was within the detection range using the flanking primers (right). **C** Visualization of Long Range PCR (LR-PCR) products on an agarose gel showing two expanded alleles in individuals #1–3

**Fig. 3:**
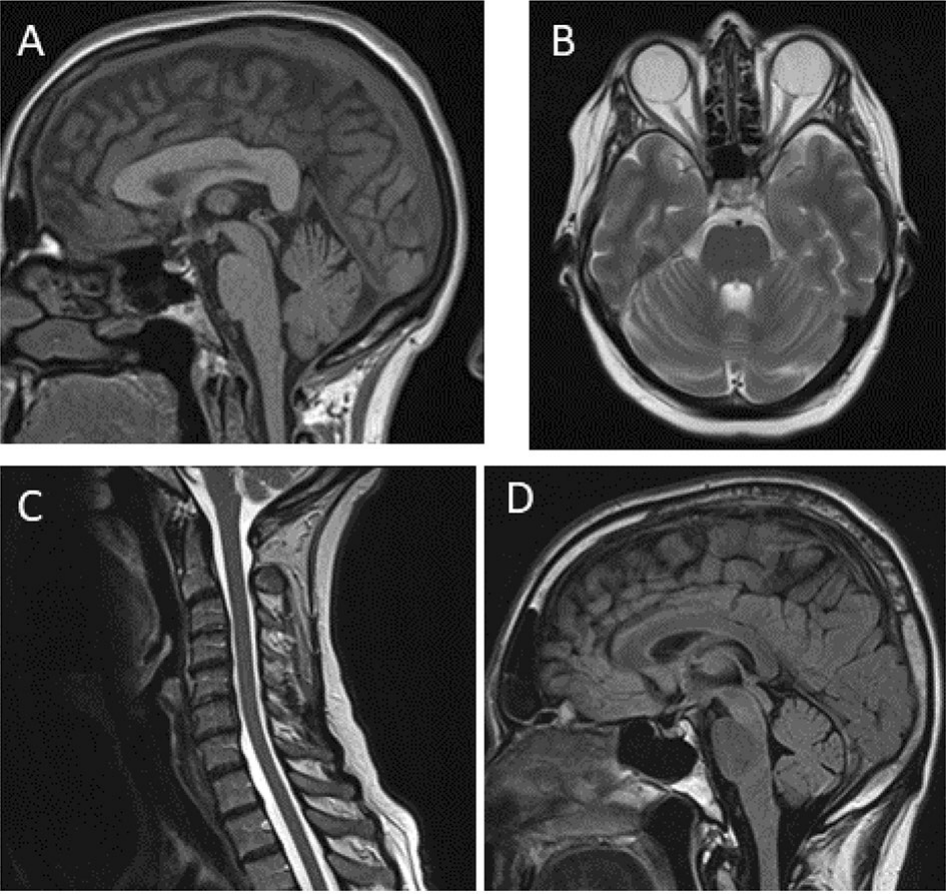
MRI sequences of Subjects #1-#3. MRI of the subjects did not show any signs of cerebellar or cervical spinal cord atrophy (exemplary sequences). **A** midsagittal T1-weighted image of cerebellum, subject #1. **B** axial T2-weighted image of cerebellum, subject #2. **C** sagittal T2-weighted image of cervical cord, subject #1. **D** midsagittal T2/FLAIR-weighted image of cerebellum, subject #3

Subject #1 is a 64-year-old female of non-consanguineous parents with a slowly progressive spastic paraparesis and cerebellar gait ataxia starting at the age of 53 years, yet without a clear afferent component. In further disease course, she developed cerebellar dysarthria, dysphagia, limb ataxia and an urge incontinence of the bladder (for detailed description, see Table 1). Muscle tendon reflexes were retained and Babinski sign was negative. Further, she showed no pes cavus or scoliosis. The family history was positive with a brother showing a similar progressive phenotype, yet also not starting before age of 57 years (Fig. 1A). The clinical work up comprised normal magnetic resonance imaging (MRI) of the brain and spinal cord (including cervical cord) without evidence of atrophies (Fig. 3A and C), and negative laboratory testing for secondary causes, including cerebrospinal fluid (CSF). After ruling out the most common PolyQ SCAs (SCA1, 2, 3, 6, 7, and 17) by direct fragment length analysis, ES was conducted revealing a variant of unknown significance (VUS) in the *ABCD1* gene (c.1084G > T: p.Ala362Ser). Consequently, X-linked Adrenoleukodystrophy (ALD)/Adrenomyeloneuropathy (AMN) was considered as the possible hereditary cause given the phenotypic compatibility of the spastic paraparesis-cerebellar ataxia phenotype (where even normal levels of very long chain fatty acids can be compatible with ALD/AMN in females) [7]. However, given that this phenotypic match was still unspecific, and the less severe phenotype in the likewise affected brother (where a more severe phenotype was to be expected in male subjects in a X-linked disease), SR-GS was performed to rule out more convincing genetic causes. It revealed a biallelic expansion in *FXN* (Fig. 2A). The exact lengths of both alleles (98/1460 GAA repeats) were determined by classical fragment length analysis and long range PCR (Fig. 2B and C), confirming the diagnosis of FA. *FXN* analysis was subsequently also performed in the affected brother by long range PCR, confirming segregation of FA repeat expansions with disease in the family.

**Table 1:** Demographic, clinical and diagnostic characteristics of the 3 subjects with atypical FA presentations identified by SR-GS

	Subject #1	Subject # 2	Subject # 3
Current age	64	59	64
Sex	F	F	M
Suspected mode of inheritance	AR	AD	AD
*FXN* GAA repeat length allele 1/allele 2	98/1460	144/650	87/1140
Age of onset [years]	53	45	49
SARA	15 at age 64y (12 at age 63y)	10 at age 59y (8.5 at age 57y; 8 at age 56y)	14.5 at age 64y (8.5 at age 58y)
Main phenotype	Spastic cerebellar ataxia	Spastic cerebellar ataxia	Spastic tetraparesis
Other neurological features (age at onset)	Reduced vibration sense, urinary urgency (60y)	Reduced vibration sense, urge incontinence	Cerebellar ataxia (49y), Reduced vibration sense (49y), urinary urgency (59y)
Electrophysiology	NA	SSEP: Median nerve normal; tibial nerve normal on the left, not evaluable on the right	Nerve conduction studies of median, ulnar, tibial and peroneal nerves normal; SSEP: Median nerve: delayed cortical latencies; tibial nerve: not evoked; MEP: Delayed latencies to arms; not evoked to legs
MRI findings	Normal (brain, cervical spinal cord, lumbar)	Normal (brain, cervical and thoracic spinal cord)	Normal (brain, cervical spinal cord)
Prior genetic diagnostics	SCA1, 2, 3, 6, 7, 17, *C9orf72*, ES	SCA1, 2, 3, 6, 7, 17, ES	SPG4, SPG5, SPG7, HSP-Panel, SCA1, 2, 3, 6, 7, ES
Relevant findings in prior genetic diagnostics	VUS c. 1084 G > T: p.Ala362Ser in *ABCD1* (ES)	no	VUS c.982C > T: p.Gln328* in *PLEKHG4* (ES)

*F* female, *M* male, *SARA* Scale for the Assessment and Rating of Ataxia [13], *AR* autosomal-recessive, *AD* autosomal-dominant, *ES* exome sequencing, *NA* not available, *SCA* spinocerebellar ataxia, *VUS* variant of unknown significance, *SSEP* somatosensory evoked potentials, *MEP* motor evoked potentials

Subject #2 is a 59-year-old female of non-consanguineous parents who developed a slowly progressive cerebellar gait disturbance with a spastic paraparesis, limb ataxia and signs of posterior column dysfunction at the age of 45 years (Table 1). She also noticed an urge incontinence of the bladder. Muscle tendon reflexes were exaggerated, while Babinski sign was negative. Pes cavus and scoliosis were absent. Laboratory tests including CSF analysis, as well as MRI of brain and spine (including cervical cord) did not provide evidence for any common secondary causes of ataxia or for atrophy of the cerebellum (Fig. 3B) or spinal cord. Family history was reported being positive with the father having developed similar gait disturbances (Fig. 1B). Under the suspicion of an autosomal dominant (AD) condition, a two-tiered genetic testing for the most common PolyQ SCAs and an ES were initiated, yielding negative results. SR-GS was added, indicating a biallelic intronic *FXN* repeat expansion (Fig. 2A), leading to the diagnosis of FA. The exact lengths of both alleles (144/650 GAA repeats) were determined by classical fragment length analysis and long range PCR (Fig. 2B and C), confirming the diagnosis of FA.

Subject #3 is a male patient of non-consanguineous parents presenting at the age of 58 years with a slowly progressive spastic tetraparesis starting at age 49 years, urge incontinence of the bladder and cerebellar signs, including limb ataxia, dysarthria, dysphagia and oculomotor deficits (Table 1). The patient showed hyperreflexia, but Babinski sign was absent, as well as pes cavus and scoliosis. Further, an involvement of the posterior column was suspected based on clinical findings and by somatosensory evoked potentials. No secondary cause was found despite extensive laboratory testing, CSF analysis and MRI of brain and spine, with MRI showing no atrophy of the cerebellum (Fig. 3D) or cervical spinal cord. A multi-tiered genetic testing was conducted including a NGS panel covering genes associated with hereditary spastic paraplegia (HSP) due to the dominant spastic phenotype. As results were negative, genetic testing for PolyQ SCAs and an ES were performed. A VUS was found in the *PLEKHG4* gene. This gene had previously been associated with an autosomal dominant (AD) pure cerebellar ataxia in several Japanese families [8]. A segregation analysis in our patient was performed, confirming the *PLEKHG4*-variant in the father, who had reportedly shown a similar gait impairment (Fig. 1C). However, given that the role of *PLEKHG4* in causing ataxia has not been fully verified and the detected VUS was also observed in the unaffected brother, SR-GS was performed to rule out more convincing genetic causes. This indicated a biallelic intronic *FXN* repeat expansion (Fig. 2A), leading to the diagnosis of FA. The exact lengths of both alleles (87/1140 GAA repeats) were determined by classical fragment length analysis and long range PCR (Fig. 2B and C), confirming the diagnosis of FA.

## Discussion

Our findings demonstrate that SR-GS allows to identify ataxia subjects with biallelic GAA expansions in *FXN*, where FA had not been part of the initial differential diagnosis, despite the fact that they were seen by ataxia experts with > 10 years of genetics ataxia experience. This was due to: atypical phenotype and atypical age of onset (subjects #1–3), misleading family history (subjects #2 and #3), and misguiding prior identification of genetic variants in phenotypically compatible ataxia genes (subjects #1 and #3). The phenotype of these subjects consisted of a late disease onset (age > 40 years in all 3 subjects) and prominent pyramidal tract symptoms, mimicking complicated hereditary spastic paraplegia (cHSP) or spastic ataxia, respectively [17]. Typical FA manifests prior to age 25 years, with a mean age of onset of 12 years [11]. The typical FA phenotype is dominated by an afferent ataxia due to a degeneration of the dorsal root ganglia and posterior columns and accompanied by cerebellar signs, whereas spasticity (if at all, rather only pyramidal weakness) is not typically part of the main presenting features or masked by a sensory polyneuropathy, in particular in early, still ambulatory disease stages. In addition, also several other typical neurological signs (absent Achilles tendon reflexes, Babinski sign) and non-neurological symptoms (e.g. scoliosis, pes cavus or hypertrophic cardiomyopathy)—characteristic of typical FA—were absent in all of these 3 subjects (as often the case in late-onset FA).

The—rather unspecific—presentation of late onset spastic ataxia, observed in all 3 subjects, makes the correct diagnosis of FA challenging, as spastic ataxias are associated with > 100 genes [17] and as FA would not be picked up by any NGS panel or ES, but require a specific pertinent genetic test for this mutation type only (direct fragment length analysis of *FXN* repeats). Against the large number of spastic ataxia gene candidates, the preselection of such a specific test—providing information on only one mutation type in one of the > 100 genes—would be in particular deprioritized and even missed if other diagnostic clinical indicators characteristic of typical FA are missing—as was the case here. Additionally, in cases #2 and #3 an autosomal-dominant genetic cause was assumed based on the respective family history, thus even delivering clinical arguments *against* direct testing for an autosomal-recessive ataxia such as FA. The genetic work up of subjects #1 and #3 entailed further misleading diagnostic information, as in both cases VUS were found in other ataxia genes that fit well phenotypically. Subject #1 harboured a VUS in the *ABCD1* gene that is associated with the X-linked recessive ALD/AMN. Given the family history with an affected brother, this condition fitted well to the clinical presentation [2] and family history. In subject #3 a VUS was discovered in *PLEKHG4* that had been associated with an autosomal-dominant form of cerebellar ataxia [8]. As this VUS was even found to co-segregate with the reportedly affected parent of subject #3, it was considered disease causing. Only the application of a hypothesis-free SR-GS and its capacity to detect intronic STRs allowed to make the correct diagnosis of FA in these subjects.

In sum, taking FA as an example, our findings illustrate how SR-GS-based diagnostics enables the correct diagnosis of an intronic STR ataxia “en route” in a phenotype- and family history-agnostic fashion, allowing to overcome clinical bias and misinformation that might be caused by: (i) atypical phenotypes and ages of onset; (ii) family history suggesting a different mode of inheritance; and (iii) prior ES-based identification of strong variants in other genes that fit well phenotypically. GS should therefore be considered early in the genetic diagnostic work up of patients with suspected hereditary neurological disorders (= “GS first approach”[1, 10, 14]), in particular in ataxias where intronic STRs are a common mutational mechanism.

## Data Availability

Data supporting the findings of our study will be available upon reasonable request to the corresponding author.
